# The Institutes of Medicine

**Published:** 1858-09

**Authors:** 


ART. III.—*The Institutes of Medicine.* By MARTYN PAINE, A.M., M.D., LL.D., Professor of the Institutes of Medicine and Materia Medica in the University of the City of New York, etc., etc.; 8vo., pp. 1095. Harper & Brothers: New York, 1858.

No one can read the volume whose title is recorded above without being filled with respect, and even admiration, for the profound erudition, the pains-taking and systematic research, and the laborious reflection exhibited so abundantly in its pages. With careful and discriminating hands, Dr. Paine has gathered together, from the writings of both the earlier and cotemporary physiologists, the numerous important facts and details which constitute the subject-matter, the crude material, so to speak, of his favorite science, and arranged and built them up into a stately edifice—the Institutiones Medicinæ—whose great corner-stones are Physiology, Pathology, and Therapeutics. Years ago the accomplishment of such a task would have required comparatively but little time and labor. At present, however, when the granaries of physiological science are fairly groaning with the golden grains of observation and experiment, gathered in by those industrious reapers and gleaners,—the experimental physiologist, the vivisector, the organic chemist and physicist, —the task has become an herculean one, demanding an extent of uninterrupted leisure, an amount of reading, and a degree of patient, persevering labor and mental application, to which few men in the medical profession can lay claim. Moreover, habits of close observation and an unusual degree of the generalizing power are qualifications essentially necessary to the undertaking. But, unfortunately, the rarity of these qualifications is equalled only by their importance. It is not to be wondered at, therefore, that the various attempts to establish a truthful, thoroughly philosophical, and satisfactory system of medical Institutes, should have proved abortive. A cursory examination of the history of physiology will suffice to show that these failures have naturally resulted from the narrow and restricted method employed in studying the operations of Nature. From time to time, as the different branches of science, under the guidance of some bold and masterly inquirer, made rapid advances in the acquisition of positive and reliable facts, Physiology received a peculiar bias in its direction. To this cause may be traced the iatro-mathematical, iatro-chemical, and other schools, each of which had its able and enthusiastic supporters, and in each of which the same fundamental error was committed—that of hasty generalization based upon isolated groups of phenomena, and the application of vague and limited laws to the explanation of the functions of the entire economy. In course of time, the array of observations and facts presented by these schools became so confused and conflicting, and appeared so incapable of elucidating function, that many physiologists, abandoning them entirely, went to the extreme of ignoring physical and chemical action altogether, and making the physics of the body wholly subservient to a mysterious force, power, or quality, which was unlike anything else in Nature, and which ruled the whole organism, seated king-like upon some special organ whence it issued its imperative mandates. Such was the vitalistic school in which the *anima* was everything; mechanics, physics, and chemistry nothing. "We are told," says Dr. Bostock, in his *History of Medicine*, "that the *anima* superintends and directs every part of the animal economy from its formation; that it prevents or repairs injuries, counteracts the effects of morbid causes, or tends to remove them when actually present, yet that we are unconscious of its existence; and that while it manifests every attribute of reason and design, it is devoid of these qualities, and is, in fact, a necessary and unintelligent agent." The majority of the physiologists of our own immediate day belong to what may be called the physico-chemico-vital school. They occupy a middle ground, between the pure vitalists and physicists of former times, assigning, with true eclecticism, some of the phenomena of life to a peculiar vital agent, and others to the action of physical forces belonging to organic in common with inorganic matter.

To this eclectic school, and to all the advocates of the chemical and physical theories of life and its manifestations, Dr. Paine is a most determined and vigorous opponent. In his Preliminary Remarks, after defining the scope of his work, explaining the legitimate objects and relations of physiology, pathology, and therapeutics, and animadverting upon the doctrines of the chemical, vital, and chemico-vital schools, he boldly throws down the gauntlet in favor of vitalism, declares himself a confirmed and thorough vitalist, discountenances, *in toto*, the labors of the modern organic chemists, and finally assails, with controversial warmth, Liebig, the great leader of the chemical school, Carpenter, Prichard, Roget, Fletcher, and others who differ from him as to the nature, properties, and relations of the vital principle. Hear him speak in his own emphatic language :—

"Chemical and mechanical philosophy, as we have already seen, are strangers to the philosophy of medicine. There is a natural conflict between the subjects of each. They have no relationship, no sympathies, but carry on a perpetual hostility. The organic being is forever converting to its own uses the inorganic, and changing its very nature into its own. The inorganic is fruitless in resistance and in assault, till the former is passive. It then lays waste the fabric by which it had been wrought into a great system of designs, and degrades the whole to its own level. Chemistry, therefore, begins where physiology ends; and physiology begins with organic influences upon the elements of matter, or where chemistry leaves off. No department of medicine has anything to hope from chemistry beyond its power of analysis.

"And yet do the labors of chemists aspire at a substitution of the ever-fluctuating principles of chemical science for all that has been hitherto founded upon the phenomena of life and disease. Their oft repeated effort to carry a science which is mainly analytical and mechanical into that which is eminently intellectual and overflowing with the most sublime institutions, and distinguished by the most profound principles and laws of Nature, and therefore seductive to an ambition which is restif under the practical manipulations of the laboratory, would raise no inquiry as to motive, or end, did not the proper guardians of the science not only abandon their old and rich domain at the very approach of the enemy, but, with most unnatural distrust of self, invite the destroyer.

"The late publication of Liebig's 'Animal Chemistry' has abundantly proved the truth of what I sufficiently established in the 'Medical and Physiological Commentaries'—that the recent application of chemistry to physiology and medicine is not a partial, but a complete substitution of that science. In justification of all this, we are now told that the means of investigation, of analysis, and of creation, have received an extension of which our predecessors had no knowledge. Such, however, has always been the pretext of chemistry for its invasions upon the science of life. Take, for example, the words of Fourcroy, who wrote more than sixty years ago, and who, like Liebig and his school, attempted to substitute chemistry for physiology, and to rear up a fabric of medicine upon that imaginary foundation; and this, too, in the case of either of the masters, without having ever read a medical book, or having ever prescribed for a disease. The language of Fourcroy is exactly such as we now hear from the lips of Liebig and his followers; who cheerfully allow that nothing flowed from the labors of Fourcroy to illuminate the dark ways of organic life. * * * * * * *

"I have said, in the Commentaries, that 'a prosperous harvest' was promised from Fourcroy's reformation. But again I reiterate, where is the evidence? since which time, also, chemistry has made greater advances than any other science, has had its unmolested sway, and Fourcroy's example has been followed with a corresponding diligence. Can you point to a solitary instance in which organic chemistry, except in a negative sense, has advanced the science of life or disease? Do not the very chemists of this day incidentally allow the perfectly abortive nature of their science in relation to physiology and medicine? Consult the quotations in section 350, etc. Or take the affirmations of the distinguished Mülder, which go, with the rest, to establish the truth of my former assertion, that '*chemistry has been a perfect incubus upon medicine; and the time is not distant when it will have proved, by its own showing, its want of relation to our subject, if it have not done so already.* * * * * * * * * * * *

"The reader should never lose sight of the foregoing hypothesis and admissions (of Liebig.) They should be ever ready to chasten his credulity as to the chemical interpretation of every organic compound. They stamp the whole 'science of organic chemistry,' in its synthetical aspects, as one of pretensions, and unworthy the confidence of an intelligent mind."

Nearly one-half of the volume before us is devoted to the consideration of physiology proper, under the various heads of the composition and structure of the human organism, the vital principle, irritability, sensibility, mobility, vital affinity, vivification, nervous power, mind, instinct, the functions, etc., together with general remarks upon the philosophy of life and death, vital habit, age, temperament, the races of men, the relations of organic beings to external objects, unity of design in physiology, etc., etc.

The details concerning the differences between organic and inorganic bodies, and the structure and composition of organic beings, a knowledge of which is necessary to a thorough comprehension of the great and important laws which guide and control the actions of man in health and disease, are all clearly and concisely set forth in a series of propositions naturally and logically successive, and almost aphorismic in their character. Nevertheless, their perspicuity is impaired, and their relation to and dependence upon each other obscured, by the polemical digressions in which the author so frequently indulges. These digressions, it is true, exhibit, to a considerable extent, the logical and argumentative powers of Dr. Paine, and show how well he has mastered the literature of his subject; but, while they render his writings racy and attractive to the advanced student who has already acquired a certain familiarity with the subject, they are, nevertheless, obnoxious to the objection that the tyro in physiology, for whose instruction the book is also intended, is led too far from his direct path, and lost in the thicket of controversy. In his really valuable remarks upon the composition of organic beings, he appears as a judge in open court, and summons before him various eminent witnesses, such as Liebig, Tiedemann, Müller, Hunter, Roget, Carpenter, Prichard, Prout, Bichat, Paget, Montgomery, and others, and cross-questions them so minutely and searchingly, that the student, dipping for the first time into this work, is like the novice in court, utterly mystified by the examination and the replies elicited. He even quarrels with the geologists about the amount of light which our globe enjoyed in the earlier periods of its existence, and hurls at them the grave charges of materialism and infidelity. This we regret to see; for although, with the thinking few, such charges, unless well sustained, fall unheeded to the ground, yet, with the thoughtless masses, who are ever as the times are, they are simply dangerous and prejudicial. In fact, whenever, in a work which professes to be purely scientific, we find such charges preferred by the author against his opponents, we are always strongly inclined to think that there is some fallacy or weak point in his argument, of which he is aware, and which he desires to conceal; and we make bold to say, that upon close examination, we have not unfrequently found such suspicion to be amply confirmed.

Our author's observations upon structure, especially in its diseased relations, will amply repay an attentive perusal. He takes strong, and we think, in the main, just ground, against the utility of the microscope in physiological research. We have long been of the opinion, and this opinion we have already freely expressed in another place, that physiology cannot expect much elucidation from minute anatomy. Nevertheless, we are by no means prepared to give our full assent to the following language, which we find on pp. 59, 60 :—

"It has already been stated, that a knowledge of the minuteness of structure which is supplied by the microscope is practically useless, while the deceptions of that instrument have led to many important errors in physiology and pathology. It cannot be depended upon, especially in exploring soft structures. If it lead to unimportant facts, it is equally liable to betray us into error and fallacious hypotheses. The whole history of that instrument, so far as physiology is concerned, has gone to confirm the foregoing conclusions, which were originally advanced in another work, and has conclusively sustained the opinion of one of the most profound observers of the present age.

"Microscopical information, so far as correct, goes to the amount of human knowledge, and to the perfection of science, though it may not contribute to useful ends. But experience shows us that we may not depend, as it respects the microscope, upon the vision of others, especially where a high magnifying power is required. Each must observe for himself; and, as allowed by Ehrenberg, long practice alone can assure him of any general accuracy. The laborious student may attend to this accomplishment. But, *vita brevis, ars longa;* and he will be likely to live the subject of deluded sense rather than of enlightened understanding.

"When we consider, therefore, the constant deceptions of the microscope, especially in all explorations of soft substances, and the absolute uselessness of any knowledge it may convey as to the recesses of organization, it may be reasonably expected that the time is not distant when all this lumber will be excluded from practical works on physiology, and turned, at least, into a channel by itself."

The third division of the physiological part of the work is occupied with the consideration of the "Properties or Powers of Life." Here it is that the positive—we are free to say too positive—vitalistic tendencies of Dr. Paine exhibit themselves in all their force.

"A vital, or peculiar governing principle or power, in organic beings, has been recognized by all the most distinguished medical philosophers at all ages of the science. It is the fundamental cause of growth, nutrition, and of all other phenomena of organic beings. It is, in all but the vulgar acceptation, synonymous with the term *life;* and *life*, therefore, is a *cause*, and not an *effect*, as has been assumed by many distinguished physiologists."

And, again, after discussing the views of the ancients and of the leading physiologists of the present day, concerning the vital principle, he proceeds:—

"*There is not, indeed, in the whole range of medical literature, one author, however devoted to the physical and chemical views of life, who does not evince the necessity of admitting a governing vital principle as a distinct entity, distinct from all other things in nature. I say, there cannot be produced one author of any consideration, who does not summon to the aid of his discussion a vital principle whenever he touches upon the abstract phenomena of life.* And this I have abundantly shown by an extensive range of quotations in my various publications."

Now, fifteen years ago, or four years before the first edition of Dr. Paine's *Institutes* was issued, there appeared in London, from the press of William Pickering, a learned work in two volumes. This work was not so much one of original research as of original reflection and extraordinary generalization; though it did not add one new fact to the sum of human knowledge, yet it embodied the results of the most laborious and protracted thought applied to the examination of all the leading and well-established facts of physical, chemical, and physiological science, with the view to harmonize such facts, to show their true relations, and to deduce from these relations a great system of natural philosophy, physiology, pathology, and therapeutics—a comprehensive, philosophical theory, in short, of the universe.

This work was spoken of by the editor of the *London Medical Gazette*, as one which "bears the unquestionable stamp of genius, and carries with it, at the same time, evidences of learning, various and extensive, and of such laborious research as can be successfully achieved only by minds of the noblest order in pursuit of sacred truth. And truly, we make bold to say, that we have never met with an effort so sustained, and we add with confidence, so successfully made, to raise the veil from the shrouded statue of Isis."* In another place, he assures us that it is a work "whose like comes from the press only at rare and distant intervals—a work, indeed, of sublime scope, and, rightly taken, of the noblest tendency. It is the truest specimen, and most successful achievement of that which the late Earl of Bridgewater had in view, when he left a sum of money for the purchase and publication of a series of treatises illustrative of the wisdom and goodness of God in creation. But it has the great advantage over any treatise which could be obtained under such circumstances, that it is not 'written to order.' It is the spontaneous effusion of the gifted mind,

* March 17, 1844, p. 229.

brimful of knowledge, and tinctured with the hallowed fire of poetry."* And again, in a foot-note at the close of his translation of *Wagner's Physiology*, Dr. Willis particularly refers to this remarkable work. The translator of Dumas and Boussingault's *Organic Chemistry* bore testimony to the originality, profundity, and comprehensiveness of its views in every branch of physical and medical science. The *British and Foreign Medical Review*, though entirely opposed, as we shall presently see, to the doctrines of the author, was forced to conclude that the "impartial and intelligent reader can feel no doubt that the foundation of his system embodies a vast extent of valuable and undoubted truths." The journals of our own country, also, lauded the book, as we might readily show by extracts therefrom, did our limited space permit. The late Dr. Caldwell, the friend and former preceptor of the author, eulogized the work most enthusiastically, while Dr. Condie, in the *American Journal of Medical Sciences*, for January, 1846, spoke of it as "evidently the production of an original mind, well versed in the natural sciences, and habituated to close and laborious investigation." (p. 133.)

Now this London book, in two volumes, which at first threatened to fall still-born from the press, and which saw the light after years of patient toil, after years of privation, suffering, and penury, cheerfully endured by its American author, that he might avail himself of the facilities for study and investigation afforded by the libraries of the British metropolis, was, after all, but the elaboration of a smaller work; an essay,† in fact, of 158 pages, read by the same author before the New York Lyceum of Natural History in 1833, and dedicated to Joseph Delafield, then president of the Lyceum. Subsequently, the views contained in this publication were somewhat extended in several spirited articles upon life, contributed by our author, while residing in New York, to the *Knickerbocker Magazine*, (1834–35.) Since the publication of "Caloric," though he wrote and studied much, we believe he published nothing more.

Need we say that the writer to whom we allude is the late Dr. S. L. Metcalfe, formerly of Kentucky, and for some years prior to his death a resident of this city, (Philadelphia.) For several years he was our neighbor and friend, with whom we were at all times happy to commune upon matters touching the science of life, and it became our painful duty at last to announce to the world, through the medium of the public papers, the news of his death, transmitted to us by telegraph, on the 17th of July,

---

* June 21, 1844.

† The reader will find a lengthy synopsis of this essay in Hinton's History and Topography of the United States. Boston, 1834. Vol. ii. p. 477.

1856, about a week after he had left the city, in the vain hope of being invigorated and restored to health by the sea-breezes of Cape Island.

This notice of Dr. Metcalfe and his writings has been introduced here as affording at once the best and most singular refutation of the statements embodied in the italicised sentences contained in the paragraphs above quoted from Dr. Paine. With the exception of the words "vital principle," the italics are our own. Indeed, it is rather remarkable that we cannot find in the *Institutes* any reference to the writings and the peculiar views of our departed friend. And yet these writings bear internal evidence that their author had been ploughing in the same great field as Dr. Paine, and, singularly enough, in the same city, and at about the same time.* These writings, moreover, employ, in a most logical manner, not a few of the same facts as those used in the *Institutes*, and many others of a wider scope, to substantiate the great fundamental principle for which their author contended most determinedly to the day of his death; the existence of a *substantive vital principle* NOT distinct from all other things in nature, but, on the contrary, identical with the *essentia caloris*; the *ουσία αἰθέρος*, or subtle fiery ether, which Pythagoras regarded as the "principle of life, animating the whole system of nature;" which Heraclitus held to be the "primordial principle of the generation of all things;" which Plato taught "to be the immediate natural agent, or animal spirit, to cherish, to warm, to enlighten, to vegetate, to produce digestion, circulation, secretion, and organic motion in all living bodies, animal and vegetable;" which Hippocrates speaks of as the "cause of motion, change, growth, diminution, etc.," and to which Newton was inclined to ascribe far more efficiency as a primal and powerful causative agent than his biographers and critics appear to be willing to admit.

In the appendix which closes his *Institutes*, Dr. Paine devotes a few pages to the discussion of the rights of authors. "Upon all questions of priority that concern the advancement of science and art," says he, "there is, doubtless, a general understanding that the principle should not only be sacredly observed, but that, whenever violated, there should be a common effort to repair the injury. This is alike due to the individual, to the principle, and to the common good." Our hearty concurrence with this opinion impels us, in connection with the above remarks, to raise here a question as to the claim of originality put forth a few years ago by Professors Grove and Carpenter, in reference to the doctrine of the correlation of the physical and vital forces.

---

* The New York Journal of Medicine for Sept. 1845, contains a review of Metcalfe's work, in which, on p. 205, distinct reference is made to the different views entertained by Drs. Metcalfe and Paine, concerning the nature of the vital principle.

Dr. Metcalfe's essay, entitled a "New Theory of Terrestrial Magnetism," and published in 1833, contains the following paragraphs, which show how far his active mind had anticipated both Grove and Carpenter in their speculations concerning the convertibility of the physical forces into each other, and the correlation of these with the vital forces.

"It will be our first object in this essay, to trace some of the most striking analogies of caloric and electricity; to show that they are radically the same subtle, imponderable, and all-pervading element; and that its unequal distribution throughout nature, is the cause of all the various powers and attractions of ponderable matter with which we are acquainted. (p. 7.) * * * * It (caloric) is the source of life and motion throughout creation. (p. 8.) * * * * We are not authorized to predict a primary distinction (between caloric and electricity) until fully acquainted with all the different states and affections of caloric under different circumstances; for example, in its combinations with different substances, in a solid, fluid, gaseous, or imponderable state—as with the matter of light—its diffusion, concentration, compression, &c. (p. 15.) * * * * There is not a greater apparent difference between any of the forms of caloric and electricity, than between the electricity in the atmosphere and in an exhausted receiver. (pp. 15, 16.) * * * * Before caloric combines with, and expands water into atmospheric vapor, it is universally acknowledged to be sensible heat; after it enters into the water and converts it into transparent invisible vapor, its state is changed; and, when greatly accumulated in this state, it exhibits electrical phenomena. To say, however, that its elementary nature is changed, would be as unphilosophical as to contend that the latent caloric of water is specifically different in its nature from the same caloric when set at liberty by pouring water on calcined lime; or that it is distinct from the caloric which moves a steam-engine by its expansion; or that the galvanic fluid is distinct from the electricity of a Leyden jar, because it moves with less velocity. (p. 16.) * * * * Had philosophers attended more carefully to the great changes which take place in the states of caloric, produced by its various *modes* of combination with other matter in different forms, they would probably have been led to discover more clearly, if not the identity of caloric and electricity, *at least that they are inseparable*, and *that without caloric there could be no electricity.* (p, 17.) * * * * One of the most decisive proofs that caloric and electricity are convertible into each other is, that during all condensation of ærial vapor, whether into rain or snow, during winter or summer, caloric is given out in very large quantities. (p. 19.) * * * * Caloric, electricity, and galvanism have hitherto constituted a separate and distinct triad of imponderables, perfectly incomprehensible; all the phenomena of which are quite intelligible, if we refer them to the agency of one grand, primary, universal element. (p. 27.) * * * * That there is a subtle, vivifying principle disseminated throughout nature, and which is intimately connected with caloric, would appear from the effect of cold on the various tribes of animal and vegetable existence. (p. 46.) * * * * One thing is certain, that innumerable forms of life spring from, or at least accompany the presence of caloric; while its absence is always attended by the entire extinction of life. Hence it would appear unphilosophical to call in the aid of some other unknown imponderable aura as a vital principle, when the agency of caloric, united (though we know not precisely in what manner) to the various forms and combinations of matter, explains the phenomena quite as well. (p. 48.) * * * * The states and affections of caloric are infinitely diversified by the various modes of its combination with ponderable matter. (p. 49.) * * * * Does not caloric answer to the subtle ether of Sir Isaac Newton? Does it not extend from the centre to the circumference of the universe? Is it not the cause of all the motions and transmutations of terrestrial matter? of decomposition and recombination? of secretion, nutrition, growth, &c.? Is it not the semperviving energy of universal nature?" (p. 52.)

These passages we have selected at random, from the first part of Dr. Metcalfe's essay, not for the purpose of claiming for him the merit of originality in the enunciation of the correlation doctrine, as applied to the physical forces; for this, as will be presently seen, we cannot accord to him; but to show that in 1833 he was not only perfectly familiar with the great doctrine in question, but contended forcibly for it in the essay above-mentioned, in which the reader will find, if he take the trouble to refer to it, not a few of the arguments employed by Grove, thirteen years afterwards, to substantiate the same great generalization. Whether Dr. Metcalfe arrived at these views independently of the writings of others, and simply by reasoning upon the well-known facts of science, we have never been able to determine; but that he was not the first to announce the correlation doctrine, we are very certain; for we have in our possession a thin octavo volume of ninety-one pages, published in Philadelphia in 1827, by Lardner Vanuxem, a Philadelphian, and entitled "An Essay on the Ultimate Principles of Chemistry, Natural Philosophy, and Physiology." On page twenty-eight of this work, we find the following clear and unmistakable proposition, the last of five conclusions which the author states to be "in conformity with the facts presented by chemistry and natural philosophy," and which he supports with abundant argument, and in a masterly manner.

"There exists but one kind of repulsive matter, as will be shown, exhibiting four different states convertible into each other, not only accordingly as it is acted upon by particles, or groups of particles forming masses, but according to the kind of particles and masses. These states of repulsive matter are caloric, light, electricity, and magnetism."

And again, on page thirty, he says:

"As all our experiments prove that caloric, electricity, magnetism, and light are convertible one into another, according to the relationships or quantities of repellant and attractive matter, it seems to me that their existence as four distinct fluids, or kinds of ethereal matter, is inadmissible; for this conversion, or change of characters, is analogous to what are called the properties of bodies, and not to the bodies themselves."

In 1837, four years after the publication of Metcalfe's essay, Dr. Samuel Jackson, the learned Professor of the Institutes of Medicine in the University of Pennsylvania, delivered and published an able Introductory Lecture, from which we quote the following paragraph :—

"Physical phenomena, according to the class they belong to, are referred to a few simple laws, as gravity, caloric, affinity, galvanism, electricity, magnetism; all of which, it can now be scarcely doubted, are modifications of one great force. The force producing physiological or organic phenomena may be no more than a modification of the same ruling power displaying its activity in organized matter."

In his Bakerian Lecture for 1845, the celebrated Professor Faraday wrote as follows :—

"I have long held an opinion almost amounting to conviction, in common, I believe, with many other lovers of natural knowledge, that the various forms under which the forces of matter are made manifest have one common origin; or in other words, are so directly related and mutually dependent, that they are convertible, as it were, one into another, and possess equivalents of power in their action. In modern times the proofs of their convertibility have been accumulated to a very considerable extent, and a commencement made of the determination of their equivalent forces."

Upon pages seven and eight of the first edition (1846) of his treatise on the "Correlation of the Physical Forces," Professor Grove thus speaks:

"The position which I seek to establish in this essay is, that the various imponderable agencies, or the affections of matter which constitute the main objects of experimental physics, viz., heat, light, electricity, magnetism, chemical affinity, and motion, are all correlative, or have a reciprocal dependence. That neither, taken abstractedly, can be said to be the essential or proximate cause of the others, but that either may, as a force, produce or be convertible into the other; thus heat may mediately or immediately produce electricity, electricity may produce heat, and so of the rest."

Here is, indeed, a remarkable coincidence, not only in ideas, but also in the language in which these ideas are clothed. The acute reader will also discover certain curious and remarkable differences and inconsistencies. These we intend to consider in detail in a future and more extended article.

In 1843, after ten years of constant application to it, Dr. Metcalfe published, in London, his larger work, entitled "Caloric, its Mechanical, Chemical, and Vital Agencies in the Phenomena of Nature." Here we find him piling fact upon fact with wonderful precision and skill, to prove that caloric, intimately combined with, and acting through organic matter as a peculiar medium, is the cause of all the complex phenomena of life; is, in fact, converted into the vital principle. Our space permits the introduction of the following quotations only :—

"The prevalent doctrine of modern physiologists, that the phenomena of life are wholly distinct from those of inorganic matter, has arisen from our imperfect knowledge in regard to the primary physical cause of motion throughout nature; and is refuted by the fact that the organizing power of the earth, like all the mechanical and chemical transformations that modify its surface, is directly in proportion to the quantity of caloric which it receives from the sun. (p. 507.) * * * * * That the power of living bodies to renew their composition by assimilation, and to reproduce their species by generation, is governed by the emphatic agency of caloric, is evident from the fact that the power of nature to multiply organic forms is directly in proportion to the temperature of the earth from the equator to the polar circles. (p. 530.) Having shown this, I proceed to prove that the organizing power of animals, and the activity of their respective functions, are directly in proportion to the quantity of the same active principle derived from the atmosphere by respiration." (p. 561.)

If the reader who may have followed us attentively will refer to page one hundred and sixty-seven of the second edition of Dr. Carpenter's "Principles of General and Comparative Physiology," published in 1841, and to page fifty-six of the first edition of his "Principles of Human Physiology," published in 1842, he will there find that Dr. C. at that time taught that "all the actions manifested by living beings are dependent upon two sets of conditions—an organized structure, possessed of certain properties which are termed *vital,* as being distinct from the physical properties of inorganic matter, and certain agents, whose presence is necessary to call these properties into operation, and thus to produce the manifestations of life. That a seed which remains unchanged during a period of many centuries, and at last vegetates, when placed in favorable circumstances, as if it had been ripened but the year before, is not *alive,* but is possessed of the property of *vitality,* or the power of performing vital actions, when aroused to them by the necessary stimuli—such as warmth, moisture, oxygen, etc. Its condition resembles that of the human being in profound sleep; he is not then a feeling, thinking man, but he is capable of feeling and thinking when he is aroused from his slumber, and his mind is put into activity by the impression of external objects."

The attention of our readers is next directed to the eighteenth article of the *British and Foreign Medical Review* for October, 1844. This article is a review of Dr. Metcalfe's work on caloric, and has been attributed, by report, to the pen of Dr. Carpenter. Indeed, we have been informed by a friend of ours, residing in London at the time to which we are now alluding, that this review was written in Bristol, by the author of several physiological works. The works of Dr. Carpenter above referred to are dated from Bristol, where he then resided. Although we cannot vouch for the truth of this report, nor the accuracy of the information, we are free to say that the style and language of the review, and the physiological ideas inculcated therein, closely resemble those of Dr. C., as will be seen from the following paragraph, which we find on page five hundred and nineteen, and which the reader is requested to compare with the passages above quoted:—

"Our readers are doubtless familiar with our views on this subject, which we may here briefly recapitulate. We hold it absurd to deny that both physical and chemical powers are at work in the living organism, and have a large share in the production of its actions; but it appears to us equally certain that there is another set of powers also concerned—those to which we give the name of *vital.* These last are exhibited only by organized tissues, possessing a certain chemical composition, and a peculiar arrangement of their structure, which no artificial means can produce; and *in all instances* the generation of a fabric capable of exhibiting vital phenomena is dependent upon the action of a previously existing organism. Now the manifestations of these phenomena depend upon certain conditions, the failure of one of which is a total preventive of them. Thus, a seed requires for its germination not only heat, but moisture and oxygen; and if the seed have lost its vitality, no amount of these other agents can excite the action in question. It appears, then, that the vital property of the seed, acting under the conditions in question, is the source of the act of germination; or perhaps it might be urged that they are all concurrent causes, equally concerned in producing the effect, because it could not take place in the absence of any one of them. But we cannot single out any one of the physical agents just alluded to as more important than the rest, since germination can no more take place without oxygen or moisture than it can without heat. Hence we deem it very unphilosophical to assert, as Dr. Metcalfe does, that caloric is *the* efficient cause of the vital actions of living beings, since it is only one of several causes which must concur to produce the results in question. And while different actions may be performed under extremely wide varieties of condition, in regard to caloric, oxygen, etc., they all agree in being immediately dependent upon those peculiar properties which an *organized* being can alone furnish."

The writer of this review calls Dr. Metcalfe an enthusiast and system-maker, accuses him of hasty generalization, and opposes *in toto* all his scientific views. On page five hundred and eighteen he expressly discountenances the attempts of Dr. M. to prove the identity of caloric and electricity, or rather that they are modifications of the same universal element; and declares that, although their analogies are strong, their differences are "still stronger, requiring that, for the present at least, their phenomena should be referred to a distinct category."

Six years after the appearance of this review, (June 20, 1850,) Dr. Carpenter read before the Royal Society a paper "On the Mutual Relations of the Vital and Physical Forces," which was published in the Philosophical Transactions of that Society. Though bearing date of 1850, the fundamental views therein promulgated were really announced by Dr. Carpenter more than two years earlier, in a review of Pareira's edition of

Matteucci's "Lectures," contributed to the *British and Foreign Medico-Chirurgical Review*, for January, 1848, p. 235. Earlier than this we have not been able to detect in the writings of Dr. C. any positive evidence of his acceptance and support of these views. On the third page of his paper just referred to, (p. 729 of the Society's volume,) he speaks of the doctrines set forth in the passages quoted above from his excellent works upon Human and Comparative Physiology, and from the review of Metcalfe's book, as the "current idea" among physiologists, and as the "doctrine of those (including himself, of course,) who have most clearly expressed themselves upon the relation of the 'vital stimuli' to the 'vital properties' of organized bodies." He declares, furthermore, that he "has not been able to find, in physiological writings, any indication of a more intimate relationship between the physical forces and vital phenomena than that just stated, save on the part of those who have vaguely identified heat or electricity with the 'vital principle,' with about the same amount of philosophical discrimination as that which was exercised by the iatro-chemists and iatro-mathematicians of the sixteenth and seventeenth centuries." We may here remark, intercurrently, that this sweeping assertion, embodying as it does a positive claim to originality, based, to a certain extent, upon a negation of the claims and labors of antecedent writers, has induced us to make this exposition, just as the singularly positive and erroneous assertion of Dr. Paine, mentioned above, led us in the first place, to refer to the writings of Dr. Metcalfe. The reviewer of Dr. M.'s work, in the early part of his article, (p. 511,) says that as "much as he respects his author's industry and zeal, and as much as he desires to serve him to the best of his ability, he respects the sacred interests of truth much more," etc. We also respect these interests, hence our present inquiry. In his valuable paper, Dr. C. proceeds to assure us that the conviction of the very intimate relationship of the physical forces has been gradually increasing in strength in the minds of philosophical inquirers during the whole of the present century, and that he regards these forces "as so many *modi operandi* of one and the same agency." The reviewer just referred to is certainly not one of these "philosophical inquirers," for, as we have just seen, he assigns the phenomena of caloric and electricity to entirely different categories. Dr. Carpenter ascribes to Grove the merit of first formularizing the "entire series of the mutual relations of these forces," and endeavors to show "that *the same relation* exists among the several vital forces, whose operation may be traced in living bodies, as exists among the physical, and that the vital and physical forces are themselves connected by a similar relationship." Finally, he urges the necessity for a certain material substratum, as the medium through which this relationship is manifested.

But the reader will find in the Essays of both Vanuxem and Metcalfe, that the importance of the medium, through which the forces act, is distinctly dwelt upon. Dr. Carpenter, in the third section of his paper, (p. 747,) tells us that the "organizing forces are so completely dependent upon the continual agency of *heat*, that they may be considered as the manifestations of the action of heat upon organized fabrics." The author of the review so frequently referred to, in the *Brit. and For. Med. Rev.* for Oct. 1844, on the other hand, writes as follows:—"That the energy and rapidity of vital action depend in great degree upon the temperature at which it takes place, is a fact which is familiar to all physiologists; and in the large amount of facts which Dr. Metcalfe has collected in support of this position, we recognize little or nothing that was not previously well known.* That caloric is the immediate and operating source of these actions, however, seems to us to be a mere hypothesis, by no means justified by the premises. We see constant indications in Dr. Metcalfe's book of his tendency to grasp at facts which support his peculiar views, and to leave all others out of consideration," etc. Now, if our readers will peruse attentively the Essay of Dr. Carpenter on the Vital and Physical Forces,

* This injudicious remark shows how incapable the writer was of appreciating the work he was reviewing. Had he read his author attentively, he would have discovered that the novelty was not in the facts, nor yet in the theory, but in the masterly and truly extraordinary manner in which Dr. M. attempted to support a leading doctrine of antiquity with the recorded facts and observations of modern science. Dr. M. troubled himself about the *accuracy* only, and not about the *novelty* of his facts. Selecting those which were long known, well established, and not to be gainsayed, he attempted to assign to them a new value and significance, by exhibiting their true and exact relations. The language which Dr. Carpenter appropriates to himself in the latter part of his Essay, applies with far more force and justice to Dr. Metcalfe. "The author has not sought," says he, "to increase the knowledge of existing *facts*, so much as to develope new *relations* between those already known. He has preferred, in fact, rather to build upon the foundation afforded by the generally admitted facts of physiological science, than to go in search of phenomena, his account of which might be questioned by those indisposed to admit his leading ideas." Had Dr. Metcalfe been less bold and decided in his views; had he constructed his work more in conformity with the style adopted in the standard text-books of Natural Philosophy and Physiology, and bestowed upon it some such name as the "Principles of Natural Philosophy, Chemistry, and Physiology, and their applications to Pathology and Therapeutics," instead of the startling and theoretical title of "Caloric," etc., we verily believe that his labors would have been crowned with success, and his book, instead of proving a failure, instead of being ignored and slighted, would have been praised and consulted. And had Dr. M. been less sensitive and dogmatic, and had he mingled more extensively and more cordially among the members of his profession, instead of shutting himself up so much in his closet, he would probably not now be sleeping in his lonely grave, the victim of a brain worn out with anxious, protracted, and thriftless study.

and compare it as carefully as we have with the earlier and later writings of Dr. Metcalfe, especially the second volume of his "Caloric," he will soon discover that, while Dr. C. and the *British and Foreign Medical Review* were industriously teaching that "vital force exists in a dormant condition in all matter capable of becoming organized," and only requires the action of certain external stimuli, such as light, heat, moisture, etc., to arouse it to activity, Dr. Metcalfe was boldly proclaiming to the world not only the close relationship of the physical forces and their dependence upon each other, but their absolute identity as modifications of one all-pervading force; not only the relationship of these to the vital forces, but also the identity of the *essentia caloris* with the *calor vitalis*, the vital principle. He will find that Dr. M. supported this proclamation, not with as little "philosophical discrimination as the iatro-chemists and iatro-mathematicians of the sixteenth and seventeenth centuries," but with all the important and well-known facts recorded in the standard, chemical, physical, and physiological works of the time in which he wrote. Finally, the reader will perceive, from the following language of Dr. Carpenter, employed on pp. 751–2 of his Essay, that, at length, seven years after the publication of Metcalfe's book, he adopts and enunciates essentially the same views. He says:—

"The views of Prof. Grove strike at the root of the notion of *latent force* of any description whatever; all force once generated being, in his estimation, perpetually *active* under one form or other; and its supposed 'latency' being a hypothetical condition, the idea of which is quite unnecessary when the force which has ceased to manifest itself is recognized under some other form. Thus, in his view, when iron is rendered magnetic by an electric current, the development of the magnetic force is rather to be looked on as the result of the conversion of the electric by the instrumentality of the iron, than as a case of the excitation of one force previously dormant, by another which is expended in thus evoking it. Such an analogy should rather lead the physiologist to look for some extraneous source of the organizing force; and to suspect that when organizable materials are applied to the extension of a living structure, and are caused to manifest vital forces, *some agency external to the organism is the moving spring of the whole series of operations.* And thus, according to the view here advocated, the vital force which causes the primordial cell of the germ first to multiply itself, and then to develope itself into a complex and extensive organism, was not either originally locked up in that single cell, nor was it latent in the materials which are progressively assimilated by itself and its descendants, but is directly and immediately supplied by the heat which is constantly operating upon it, and which is transformed into vital force by its passage through the organized fabric that manifests it. The facts already cited, which show how completely dependent the process of germ-development, both in plants and animals, is upon the constant agency of heat, and how precisely its rate may be regulated by the measure of that force supplied to it, appear to the author to be so much better accounted for upon this view than upon either of the others, that he ventures to think that they demonstrate it almost as fully as the nature of physiological evidence will admit.

"Having thus contrasted the doctrine for which he is contending with those which are current among physiologists, the author thinks it well to point out that he no more regards heat as the 'vital principle,' or as itself identical with the 'vital force,' than it is identical with electricity or with chemical affinity. Nor does he in the least recognize the possibility that any action of heat upon the inorganic elements can of itself develope an organized structure of even the simplest kind. The pre-existence of a living organism, through which *alone* can heat be converted into vital force, is as necessary upon this theory, as it is upon any of those currently received among physiologists. And it is the *speciality* of the material substratum thus furnishing the medium or instrument of the metamorphosis, which in his opinion establishes, and must ever maintain, a well-marked boundary-line between the Physical and the Vital forces. Starting with the abstract notion of Force, as emanating at once from the Divine Will, we might say that this force, operating through inorganic matter, manifests itself in electricity, magnetism, light, heat, chemical affinity, and mechanical motion; but that, when directed through organized structures, it effects the operations of growth, development, chemico-vital transformation, and the like; and is further metamorphosed, through the instrumentality of the structures thus generated, into nervous agency and muscular power. If we *only* knew of heat as it acts upon the organized creation, the peculiarities of its operation upon inorganic matters would seem as strange to the physiologist as the effects here attributed to it may appear to those who are only accustomed to contemplate the physical phenomena to which it gives rise."

Now, it is very evident, from this quotation, that Dr. Carpenter believes,—1. That there is no such thing as a latent or dormant vital principle. 2. That "the moving spring of the organic operations is some agency external to the organism." 3. That heat is, in fact, that agency. 4. That heat is something more than a mere stimulus. 5. That "heat is transformed into vital force by its passage through an organized fabric." 6. That the peculiar material substratum in which this transformation takes place is the "medium or instrument of the metamorphosis." From these premises the conclusion is irresistible that heat and the vital force or principle are identical. And yet Dr. C., remarkably enough, expressly declares that he considers heat and the vital principle as distinct forces. This looks to us very much like trifling with both language and truth. It is saying one thing to the ear, and another to the mind. It is holding two doctrines, like the ancient philosophers—one esoteric, for the learned few, the other exoteric, for the ignorant many. In view of these statements, are we not forced to conclude that Dr. C. is either very uncertain about the correlative theory, seeing it through a glass darkly, or, having a wholesome dread of the "odium theologicum," he is unwilling to brave openly the unenlightened and mistaken prejudices of those who are so bitterly opposed to all the physical theories of life and organization?

In concluding these remarks, we must say, in justice to ourself, that we have been actuated in making this exposition purely by our love for "fair play and authors' rights." We have the highest respect for Dr. Carpenter and his well-deserved reputation as a physiologist. For a number of years we have consulted his various excellent works on physiology with equal delight and profit, and we have constantly recommended these works to our students as able and well-digested compendia of the science.

The history of the rise and growth of the doctrine of correlation is a very curious and interesting one. We have devoted considerable attention to it, and, in the foregoing remarks, have anticipated a paper on the "Physical and Teleological Theories of Life and Organization," which we have been long preparing, but which our manifold duties have thus far prevented us from completing. In that paper we purpose to analyze critically the correlation theories of Vanuxem, Grove, and Carpenter, and to notice particularly the doctrines set forth in Mayer's Treatise upon "Organic Movements in their Relations to Material Changes," (1845;) Trastour's pamphlet, entitled "Caloric, Origin, Matter, and Law of the Universe," (1847;) the essay on the "Identities of Light and Heat, of Caloric and Electricity," (1848,) from the pen of our esteemed friend, Dr. C. C. Cooper; Burnett's "Philosophy of Spirits in Relation to Matter," (1850;) Radcliffe's "Philosophy of Vital Motion," (1851;) and the elaborate volume of Z. Allen, on the "Philosophy of the Mechanics of Nature," (1852,) with which, and other kindred works, we have long been familiar. In the mean time, we earnestly hope that our distinguished friend, the American editor of Carpenter's "Human Physiology," will, in the next reprint of that work, do justice to the scientific claims of Vanuxem and Metcalfe, as he has already emphatically done to those of the present eminent incumbent of the chair of physiology in the University of Pennsylvania.

Dr. Paine dwells at considerable length upon the vital principle, examining carefully and profoundly its various properties; its remarkable analogies with the soul and with the principle of instinct; the history of its vicissitudes with medical philosophers; the different opinions advanced from time to time concerning it; the phenomena by which its existence and laws are adequately attested; its nature and its inseparability from, and relation to, living organic matter. He discusses its indivisibility, mutability, and creative power; contends that it is essentially the same in plants and animals; is independent of chemical action; resists chemical agencies; presides over organic processes and results; and is, in fact, the fundamental cause of all the phenomena of organic beings. He thinks, also, that its mutability, though designed for useful purposes, is the essential cause of disease; that its nature has been altered in man since his creation; that it is subject to extinction, and becomes its own destroyer in virtue of its formative action, and that it is a bond of union between mind and matter. He attempts, furthermore, to explain how it combines the elements of matter in plants; how it modifies and appropriates organic compounds in animals, and rearranges their elements; how far it is creative; how it influences growth, and the development of the germ, and generates motion.

The second division of the *Institutes* is devoted to Pathology; under which head, Dr. P. treats of remote and pathological or proximate causes, symptoms, morbid anatomy, inflammation, fever, venous congestion, humoralism, etc. The third part, consisting of nearly 250 pages, is an extended treatise upon Therapeutics.

Dr. Paine is an extreme solidist. Disease of the fluids is with him invariably the result of diseased solids. He rejects as inaccurate and useless all the chemical analyses of the blood and fluids which the chemists have, from time to time, placed upon record. He denies the possibility of the blood becoming a cause of disease, and ascribes the abnormal states of this fluid to deranged conditions of the nervous system. In the direct and reflex actions of the nervous system he finds an explanation of all the phenomena of pathology and therapeutics. He disbelieves in the direct action of drugs upon the blood, and thinks that their influence is exerted upon the solids, and that remedies operate like morbific causes, giving rise to a series of pathological conditions.

A valuable appendix, in which, among other topics, are discussed the progress of physiological and pathological chemistry, absorption and circulation in plants, the possibility of artificially reducing the quantity of blood circulating in the brain, etc., and two very copious and systematic indices of 175 pages, complete the volume before us.

However much we might desire to do so, our limits, already transgressed, prevent us from giving our readers a complete analysis of the peculiar views of Dr. Paine. We hasten, therefore, to conclude our remarks by earnestly recommending his work to the careful perusal and study of every one interested in physiology, whether in its aspect as a pure or an applied science. Though it contains many inaccuracies of detail, and much that is vague and merely controversial, yet the breadth and comprehensiveness of many of its doctrines, the great questions in which it abounds, and the consummate skill and learning with which these are generally treated, stamp it as a valuable treatise which should find a place in every philosophical library, and be consulted by every physician who practices his profession as a science and not as an empirical art.

